# Metagenomic insights into the relationship between gut microbiota and residual feed intake of small-sized meat ducks

**DOI:** 10.3389/fmicb.2022.1075610

**Published:** 2023-01-18

**Authors:** Hao Bai, Lei Shi, Qixin Guo, Yong Jiang, Xiaofan Li, Dandan Geng, Chenxiao Wang, Yulin Bi, Zhixiu Wang, Guohong Chen, Fuguang Xue, Guobin Chang

**Affiliations:** ^1^Joint International Research Laboratory of Agriculture and Agri-Product Safety, The Ministry of Education of China, Yangzhou University, Yangzhou, China; ^2^Nanchang Key Laboratory of Animal Health and Safety Production, Jiangxi Agricultural University, Nanchang, China; ^3^College of Animal Science and Technology, Hebei Agricultural University, Baoding, China

**Keywords:** small-sized meat ducks, residual feed intake, gut microbiota, feed efficiency, carbohydrate metabolism

## Abstract

**Introduction:**

The objective of this study was to determine the regulatory effects of gut microbiota on the feed efficiency (FE) of small-sized meat ducks by evaluating correlations between gut microbiota and residual feed intake (RFI).

**Methods:**

A total of 500 21-day-old healthy male ducks with similar initial body weights (645 ± 15.0 g) were raised contemporaneously in the same experimental facility until slaughter at 56 days of age. In total, nine low-RFI (LR) and nine high-RFI (HR) birds were selected for further gut microbiota composition and functional analyses based on the production performance, and the RFI was calculated from 22 to 56 days of age.

**Results:**

Growth performance results indicated a significantly lower RFI, feed conversion ratio, feed intake, and average daily feed intake in the LR ducks (*P* < 0.05). Taxonomy results of gut microbiota showed the identification of 19 kinds of phyla and more than 250 kinds of genera in all samples. No significant discrepancies in cecal bacterial α-diversity were discovered between the LR and HR groups, which indicated that the microbial modulatory effects on RFI may be attributed to the bacterial composition, rather than the species diversity. Differential analysis of bacterial communities between the LR and HR groups showed a significant increment of *Firmicutes* and a significant decline of *Bacteroidetes* in the LR group (*P* < 0.05). Specifically, genera of *Erysipelatoclostridium, Parasutterella, Fournierella*, and *Lactococcus* significantly proliferated, while *Bacteroides* significantly decreased in the LR group (*P* < 0.05). Furthermore, correlation analysis showed that the RFI was significantly correlated with carbohydrate metabolism-related bacteria including *Bacteroides, Alistipes, Bifidobacterium, Ruminiclostridium_9, Sellimonas, Oscillibacter, Escherichia-Shigella, Lactococcus*, and *Streptococcus*.

**Conclusion:**

In conclusion, the communities related to carbohydrate metabolism had positive regulatory effects on the FE of small-sized meat ducks, promoting it by improving the relative abundance and utilization of these communities. The present study provides valuable insight into the dynamics of gut microbiota underlying the variations in the FE of small-sized meat ducks.

## Introduction

The husbandry industry has contributed to significant improvements in the feed efficiency (FE) by using both genetic and non-genetic methods to reduce cost input and to increase the sustainability of animal production (Calderón Díaz et al., 2017; Cantalapiedra-Hijar et al., 2018; Lee et al., 2021). Traditional genetic selections mainly referred to body size, and those animals with larger body sizes were reserved. However, animals with similar body weights (BWs) always required rather widely different amounts of feedstuff for similar body weight gain (BWG) (Yi et al., 2018). Moreover, the accurate calculation of the nutritional requirement of growing animals was far beyond the directed ratio calculation between the feed intake (FI) and BWG (Kelly et al., 2019; Poompramun et al., 2021a). Therefore, the residual feed intake (RFI), which indicates the discrepancy between the actual and the predicted FI based on the body size and growth rate of animals, was applied to improve the animal FE (Bezerra et al., 2013).

Despite the favorable FE of fast-growing meat ducks, such as Pekin ducks and Cherry Valley ducks, a lower FE seriously restricts the production of small-sized meat ducks, requiring a critical enhancement of feed utilization to reduce cost inputs. Previous studies indicated an effective improvement in broiler FE by the genetic selection of lower RFI, which resulted in the reduction of the FI and abdominal fat content without affecting the BW and intramuscular fat content (Yang et al., 2020; Poompramun et al., 2021b). Intriguingly, our previous study confirmed the benefits of low-RFI selection on improving the FE of small-sized meat ducks without affecting their BWG, marketing BW, carcass composition, and meat quality (Bai et al., 2022). Further investigation showed that a series of bacterial features were differentially enriched between high- and low-RFI chickens (Liu et al., 2021). However, how these bacteria work and deeper insights into the potential regulatory mechanism of the RFI of ducks were still lacking.

The gut microbiome is considered the host second genome and has emerged as a key determinant of the enhancement of feed digestibility and nutrient absorption in the digestive tract (Wen et al., 2021). Unlike other environmental factors, the microbiome may be transmitted between generations, carrying the potential to alter traits beyond the limits of the host genetics (Jackrel et al., 2020). Feed efficiency may be modulated by certain representative gut bacteria by increasing the bioavailable nutrient pools (Jackrel et al., 2020), and therefore, we hypothesized that the gut microbiota community may also exhibit potential regulatory effects on the RFI, and bacterial communities that possessed a higher starch degradability and energy generation positively correlated with a lower RFI and significantly increased the FE.

## Materials and methods

### Animals and experimental design

A total of 600 1-day-old male small-sized meat ducks (H strain) were procured from Ecolovo Group, China. All the ducks were raised on the floor (15 birds/m^2^) during the first 3 weeks. Ducks with the highest and lowest BWs and those that died or had leg problems were excluded at 21 days of age (*n* = 100). The remaining 500 birds, with similar initial body weights (645 ± 15 g), were then transferred to individual cages (73 × 55 × 80 cm), with feed and water provided *ad libitum*. All the birds were raised contemporaneously in the same experimental facility until slaughter at 56 days of age. The composition and chemical ingredients of the diet are shown in Table 1.

**Table 1:** Compositions of and nutrients in the experimental diets.

**Item**	**0–21 days**	**22–56 days**
**Ingredient (%)**		
Corn	10.32	21.27
Wheat middling	15.41	20.00
Wheat bran	-	30.01
Rice noodles	35.21	10.00
Rice bran	15.81	5.00
Peanut meal	-	2.37
Soybean meal	12.63	2.50
Nucleotide slag	2.00	-
Limestone powder	1.52	1.96
Calcium hydrogen phosphate	1.10	0.89
Compound premix^a^	6.00	6.00
Total	100	100
**Formulated nutrient profile (g/kg)**		
Crude protein	210.00	140.00
Crude fat	20.00	35.00
Crude fiber	50.00	70.00
Crude ash	70.00	100.00
Calcium	10.00	10.00
Phosphorus	6.00	4.50
Sodium chloride	6.00	6.00
Methionine	4.00	2.80
Moisture	140.00	140.00

a Supplied per kilogram of total diet: bentonite, 44.46 g; lysine, 3.24 g; DL-MHA-FA (88%), 0.99 g; threonine, 0.73 g; sodium chloride, 4.40 g; sodium bicarbonate, 2.00 g; sodium sulfate, 2.00 g; herbalife, 0.20 g; choline chloride (60%), 1.00 g; C-811 enzyme, 0.30 g.

Vitamin content: VA 12,000 IU/kg; VD 33,000 IU/kg; VE 7.5 IU/kg; VK 31.50 mg/kg; VB1 0.6 mg/kg; VB2 4.8 mg/kg; VB6 1.8 mg/kg; VB12 10 mg/kg; folic acid 0.15 mg/kg; niacinamide 30 mg/kg; pantothenic acid 10.5 mg/kg.

Mineral content: Fe 80 mg, Cu 8 mg, Mn 80 mg, Zn 60 mg, Se 0.15 mg, I 0.35 mg.

### Determination of growth performance

The final BW and feed consumption were weighed for each bird that received a 12-h-long fasting treatment. Growth performance parameters including metabolic body weight (MBW^0.75^), BWG, FI, average daily weight gain (ADG), average daily feed intake (ADFI), and feed conversion ratio (FCR), were then calculated based on the results recorded from 22 to 56 days of age. The RFI was calculated following the method described by Aggrey et al. (2010). The equation used is provided as follows:


$$\begin{array}{l} \text{RFI}=\text{FI}-\left(a+b_{1}\times {\text{MBW}}^{0.75}+b_{2}\times \text{ADG}\right) \end{array}$$

where *a* represents the intercept, *b*_1_ represents the regression coefficients of FI on MBW^0.75^, and *b*_2_ represents the regression coefficients of FI on ADG. The RFI values were calculated following the REG procedure in SAS (version 9.4, SAS Institute Inc., Cary, NC, USA).

### Cecal content sampling and microbiota sequencing

A total of 18 birds, including nine low-RFI (LR) and nine high-RFI (HR) birds, based on the calculation results, were selected for cecal content sample collection. Samples from each bird were collected in a sterile tube, followed by a rapid freezing treatment with liquid nitrogen, and stored at −80°C for further analysis. DNA from each sample was extracted using the cetyltrimethylammonium bromide and sodium dodecyl sulfate (CTAB/SDS) method described by Wang et al. (2012). The concentration and purity were then measured to ensure the availability of each DNA sample. Following that, the V4 region of the 16S rRNA gene was amplified using the universal primers 520F (F: GTGCCAGCMGCCGCGGTAA) and 802R (R: GGACTACHVGGGTWTCTAAT). Electrophoresis samples, with a bright main strip between 400 and 450 bp, were selected for further sequencing analysis. The PCR products were purified using a Qiagen Gel Extraction Kit (Qiagen, Hilden, Germany), followed by the generation of sequencing libraries using a TruSeq^®^ DNA PCR-Free Sample Preparation Kit (Illumina, USA). The library was sequenced on an Illumina Novaseq 6000 platform (Illumina Inc., San Diego, USA) after assessment of the library quality using a Qubit@ 2.0 Fluorometer (Thermo Scientific) and an Agilent Bioanalyzer 2100 system. Quality filtering of raw tags was performed under specific filtering conditions (length < 50 bp, or with a quality value of < 20, or having N bases) to obtain the high-quality clean tags in accordance with the Quantitative Insights Into Microbial Ecology (QIIME, V1.7.0) quality-control process. Sequences with a similarity of > 97% were assigned to the same operational taxonomic unit (OTU). For each representative sequence, the Greengenes database was used, based on the SILVA classifier algorithm, to annotate taxonomic information. All taxonomic results were then further processed by differential analysis of the bacterial community of the low- and high-RFI small-sized meat ducks.

### Differential analysis of cecal microbiota

Relative OTU abundances of each sample were calculated based on the normal distribution test, and then the independent Student's *t*-test of SAS 9.4 (version 9.4, SAS Institute Inc., Cary, NC, USA) was applied for the differential analysis. Alpha diversity and beta diversity of our samples were calculated by QIIME (version 1.7.0) and displayed using R software (version 4.1.3, R Core Team, Vienna, Austria). Principal component analysis (PCA) was performed using the ggplot2 package in R software. Pearson's correlations between bacterial communities and production performance were assessed using the PROC CORR procedure in SAS 9.4, and then the correlation matrix was created and visualized in a heatmap format by using R software.

### Statistical analysis

The RFI values were calculated using the REG procedure in SAS (version 9.4, SAS Institute Inc., Cary, NC, USA). Differential analysis of growth performances between the low and high RFI was first verified by using the normal distribution test using the SAS procedure “proc univariate data=test normal,” followed by the independent Student's *t*-test to investigate the differences between the low- and high-RFI birds. The results were presented as mean ± SEM, and a *p* < 0.05 was considered significant, and 0.05 ≤ *P* < 0.10 was considered a tendency.

## Results

### Growth performance

The effects of RFI divergence on the growth performance of small-sized meat ducks are shown in Table 2. The FI and ADFI of the birds in the LR group (5797.50 and 165.60 g, respectively) were found to be approximately 26.4% lower than those in the HR (7878.20 and 225.10 g, respectively) group (*P* < 0.01). Significant differences (*P* < 0.01) were observed in the RFI and FCR between the LR (−22.22 and 4.28 g) and HR (32.96 and 5.67 g) groups, respectively. Importantly, no differences were detected in the initial BW, final BW, MBW^0.75^, BWG, and ADG between the two groups (*P* > 0.05).

**Table 2:** Effects of RFI divergence on the growth performance of small-sized meat ducks from 22 to 56 days of age.

**Items**	**LR**	**HR**	**SEM**	***P*-value**
Initial BW (g)	633.30	655.00	46.744	0.653
Final BW (g)	1988.70	2044.70	118.209	0.646
MBW^0.75^ (g)	297.50	303.80	13.280	0.646
BWG (g)	1355.30	1389.70	73.391	0.650
ADG (g)	38.70	39.70	2.108	0.650
FI (g)	5797.50^b^	7878.20^a^	344.850	< 0.001
ADFI (g)	165.60^b^	225.10^a^	9.852	< 0.001
FCR (g/g)	4.28^b^	5.67^a^	0.041	< 0.001
RFI (g/d)	−22.22^b^	32.96^a^	4.316	< 0.001

^a, b^Within a row for each item, different superscripts indicate significant differences (*P* < 0.05).

LR, low residue feed intake; HR, high residue feed intake; SEM, standard error of mean.

Initial BW, body weight on day 22; final BW, body weight on day 56; MBW^0.75^, metabolic body weight on day 56; BWG, body weight gain; ADG, average daily gain; FI, feed intake; ADFI, average daily feed intake; FCR, feed conversion ratio; RFI, residual feed intake.

### Cecal microbiota

**Figure 1:**
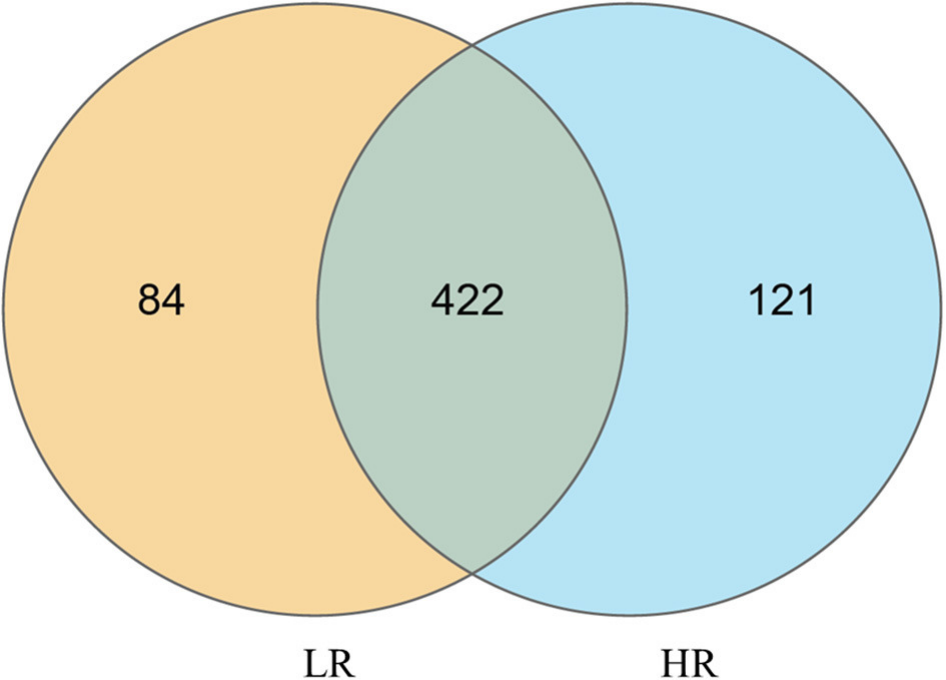
Quantitative analysis of the differential OTUs between low- and high-residual feed intake small-sized meat ducks.

16S rRNA gene amplicon sequencing analysis of the discrepancies between LR and HR ducks was conducted to identify the critical gastrointestinal microbiota. Taxonomy results of all bacteria are shown in Supplementary Table 1. In total, 19 kinds of phyla and more than 250 kinds of genera were identified in the present study, after data filtering. All the results were subsequently used for further differential and functional analyses. An integral insight into the differential OTUs between LR and HR groups using Venn diagrams was first conducted, and the results are shown in Figure 1. Based on the results, 422 of the identified OTUs were clustered in both LR and HR groups. HR ducks processed 121 unique OTUs, while LR processed 84 unique OTUs. All these OTUs were selected for the following α-diversity and β-diversity analyses.

#### α-diversity

Alpha diversity was first applied to analyze the complexity of species diversity using Sobs, Chao, Shannon, Simpson, ACE, pielou, and pd indexes. All the results are displayed in Table 3. The results showed that no significant discrepancies in the cecal bacterial diversity were discovered between LR and HR groups (*P* > 0.05), which indicates that the modulatory effects of microbial communities on the RFI can be mainly attributed to the bacterial community composition, rather than the species diversity.

**Table 3:** Effects of RFI divergence on α-diversity of small-sized meat ducks.

**Items**	**LR (*n* = 9)**	**HR (*n* = 9)**	**SEM**	***P*-value**
Sobs	524.3	548.4	27.66	0.434
Shannon	4.84	4.90	0.341	0.855
Simpson	0.90	0.90	0.024	0.960
Chao	647.6	663.9	22.84	0.516
Ace	666.2	696.5	28.24	0.343
Pielou	0.54	0.54	0.033	0.925
Pd	59.17	63.03	3.327	0.310

LR, low residual feed intake; HR, high residual feed intake; SEM, standard error of mean.

#### β-diversity

**Figure 2:**
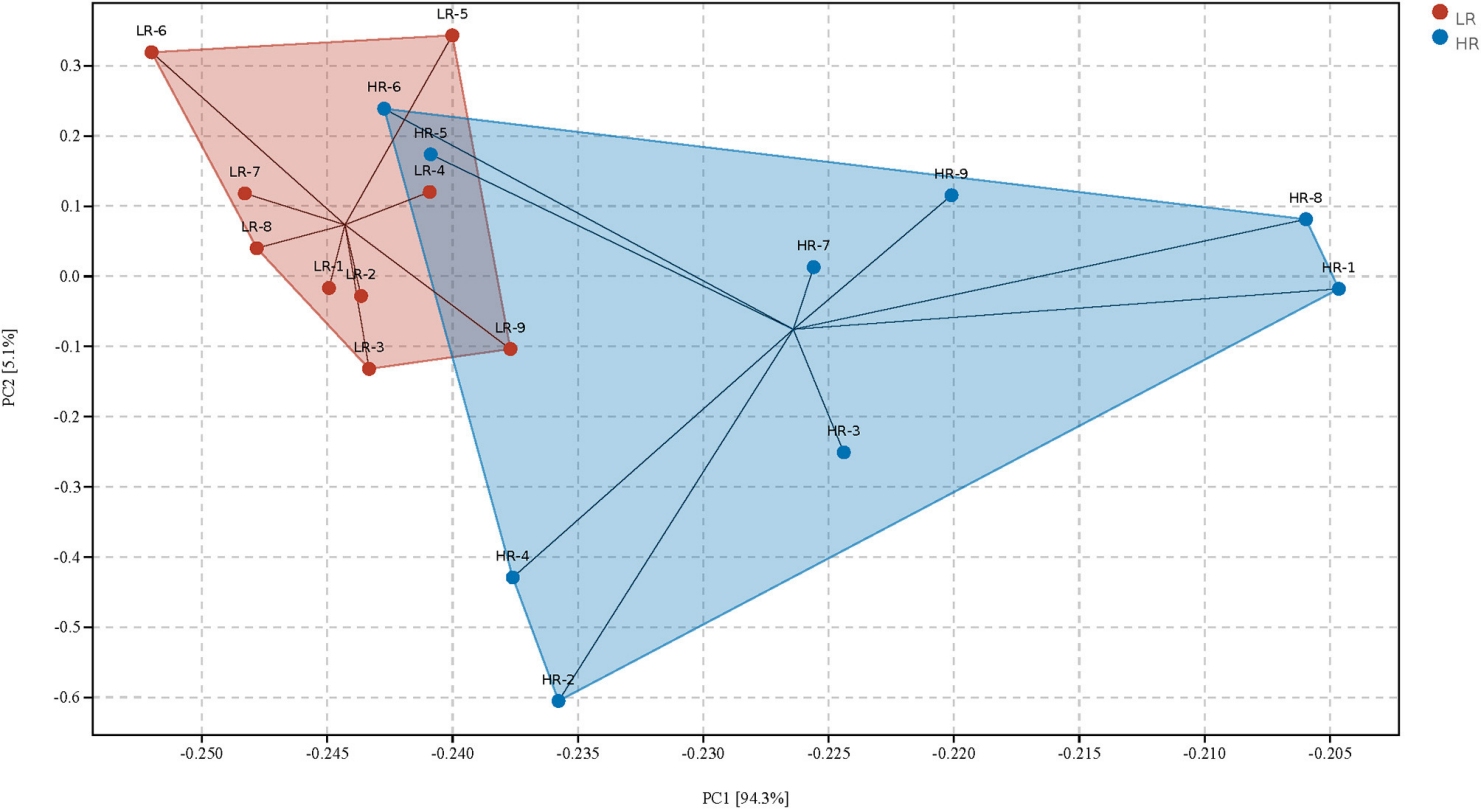
Principal component analysis of the gut microbiota community structures between high- and the low-residue feed intake ducks. LR, low residual feed intake; HR, high residual feed intake.

PCA was first performed to clarify the monolithic discrepancy of bacterial profiles between LR and HR ducks, and the result is shown in Figure 2. PC1 and PC2 accounted for 94.3 and 5.1% of the total discrepancy, respectively. Bacterial communities within LR and HR birds showed a clear separation from each other, except HR5 and HR6. This result indicated a significant alteration of bacterial communities between LR and HR groups; thus, further differential analysis of the two groups is required. Differential analysis of the relative bacterial abundances between LR and HR groups at both phyla and genera levels was conducted, and the results are shown in Tables 4, 5. As shown in Table 4, *Firmicutes* and *Bacteroidetes* were the top two items in the biomass of the total microbiota and were significantly altered on account of the change in RFI. *Firmicutes* significantly increased in the LR ducks (*P* < 0.05), while *Bacteroidetes* presented a completely inverse alteration in the LR ducks (*P* < 0.05). No significant alterations were observed in the remaining phyla (*P* > 0.05). Furthermore, a differential analysis of the bacterial communities at the genus level was conducted, and the results are shown in Table 5. *Bacteroides, Alistipes, Megamonas, Barnesiella*, and *Faecalibacterium* were the five most abundant bacterial communities and contributed to almost 70% of the total bacterial biomass. *Erysipelatoclostridium, Parasutterella, Fournierella, Blautia*, and *Lactococcus* were significantly proliferated, while *Bacteroides* significantly decreased in the LR group (*P* < 0.05). No other genera were significantly altered between the LR and HR treatments (*P* > 0.05).

**Table 4:** Effects of RFI divergence on the relative abundances of small-sized meat ducks (%, level of phyla).

**Items**	**LR**	**HR**	**SEM**	***P*-value**
*Bacteroidetes*	42.27^b^	52.79^a^	3.249	< 0.001
*Firmicutes*	50.37^a^	37.51^b^	4.298	0.009
*Proteobacteria*	3.53	2.38	1.470	0.816
*Verrucomicrobia*	2.88	5.09	1.737	0.156
*Actinobacteria*	0.29	1.97	0.369	0.280
*Cyanobacteria*	0.14	0.11	0.060	0.584
*Tenericutes*	0.22	0.08	0.118	0.164
*Epsilonbacteraeota*	0.02	0.01	0.011	0.093
*Patescibacteria*	0.01	0.01	0.001	0.551
*Chloroflexi*	0.01	0.01	0.001	0.853
*Acidobacteria*	0.01	0.01	0.001	0.715
Others	0.32	0.07	0.011	0.073

^a, b^Within a row for each item, different superscripts indicate significant differences (*P* < 0.05).

LR, low residual feed intake; HR, high residual feed intake; SEM, standard error of mean.

**Table 5:** Effects of RFI divergence on the relative abundances of small-sized meat ducks (%, level of genera).

**Items**	**LR**	**HR**	**SEM**	***P*-value**
*Bacteroides*	29.04^b^	38.13^a^	1.455	0.008
*Alistipes*	20.57	18.07	1.432	0.563
*Megamonas*	9.24	4.93	1.509	0.435
*Barnesiella*	3.12	3.67	1.114	0.705
*Faecalibacterium*	3.03	3.68	0.886	0.562
*Phascolarctobacterium*	2.47	4.01	0.573	0.102
*Eubacterium_coprostanoligenes_group*	3.06	2.92	0.435	0.887
*Ruminococcus_torques_group*	2.98	2.31	0.382	0.443
*Erysipelatoclostridium*	2.82^a^	1.25^b^	0.345	0.002
*Ruminococcaceae*	1.38	2.68	0.747	0.128
*Parasutterella*	2.21^a^	0.48^b^	0.161	0.002
*Desulfovibrio*	0.22	1.59	0.865	0.144
*Fournierella*	1.20^a^	0.45^b^	0.115	0.005
*Subdoligranulum*	0.84	0.65	0.161	0.540
*Bifidobacterium*	0.10	1.33	0.666	0.095
*Parabacteroides*	0.52	0.72	0.185	0.362
*Ruminiclostridium_9*	0.87	0.31	0.395	0.184
*GCA-900066225*	0.62	0.47	0.205	0.517
*Sellimonas*	0.67	0.41	0.058	0.078
*Oscillospira*	0.65	0.42	0.073	0.223
*Blautia*	0.77^a^	0.24^b^	0.079	0.018
*Butyricicoccus*	0.39	0.39	0.123	0.973
*Oscillibacter*	0.48	0.24	0.193	0.252
*Escherichia-Shigella*	0.49	0.17	0.250	0.252
*Lactococcus*	0.03^a^	0.01^b^	0.004	0.011
*Lactobacillus*	0.01	0.01	0.001	0.300
*Streptococcus*	0.01	0.01	0.004	0.795
Others	3.99	2.96	0.023	0.073

^a, b^Within a row for each item, different superscripts indicate significant differences (*P* < 0.05).

LR, low residual feed intake; HR, high residual feed intake; SEM, standard error of mean.

### Functional prediction of the differential gut microbiota

**Figure 3:**
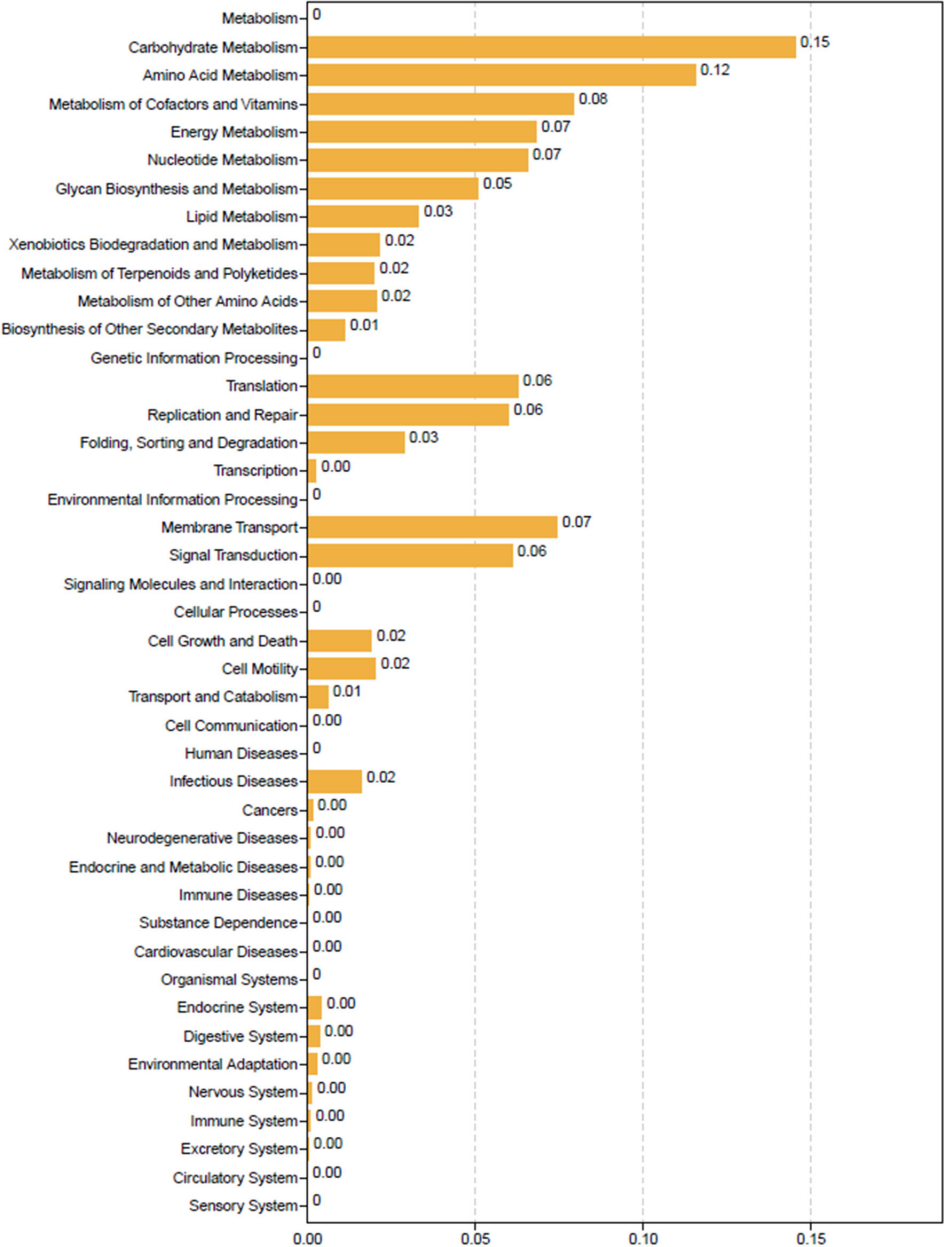
Tax4Fun functional prediction analysis of the differential abundant bacterial communities between low-and high-residual feed intake ducks.

For functional prediction analysis, the differentially identified gut microbiota of LR and HR ducks were identified using Tax4Fun (Aßhauer et al., 2015), and the result is shown in Figure 3. Metabolism processes, which included carbohydrate metabolism, amino acid metabolism, energy metabolism, cofactor and vitamin metabolism, and lipid metabolism, were the most abundant functions enriched from differential bacteria. Specifically, the biosynthesis of other secondary metabolites played an important role in the nutrient digestion process and also enriched the result. Other functions including cell viability and immune diseases were slightly correlated with the RFI.

### Correlations between the gut microbiota and growth performance

**Figure 4:**
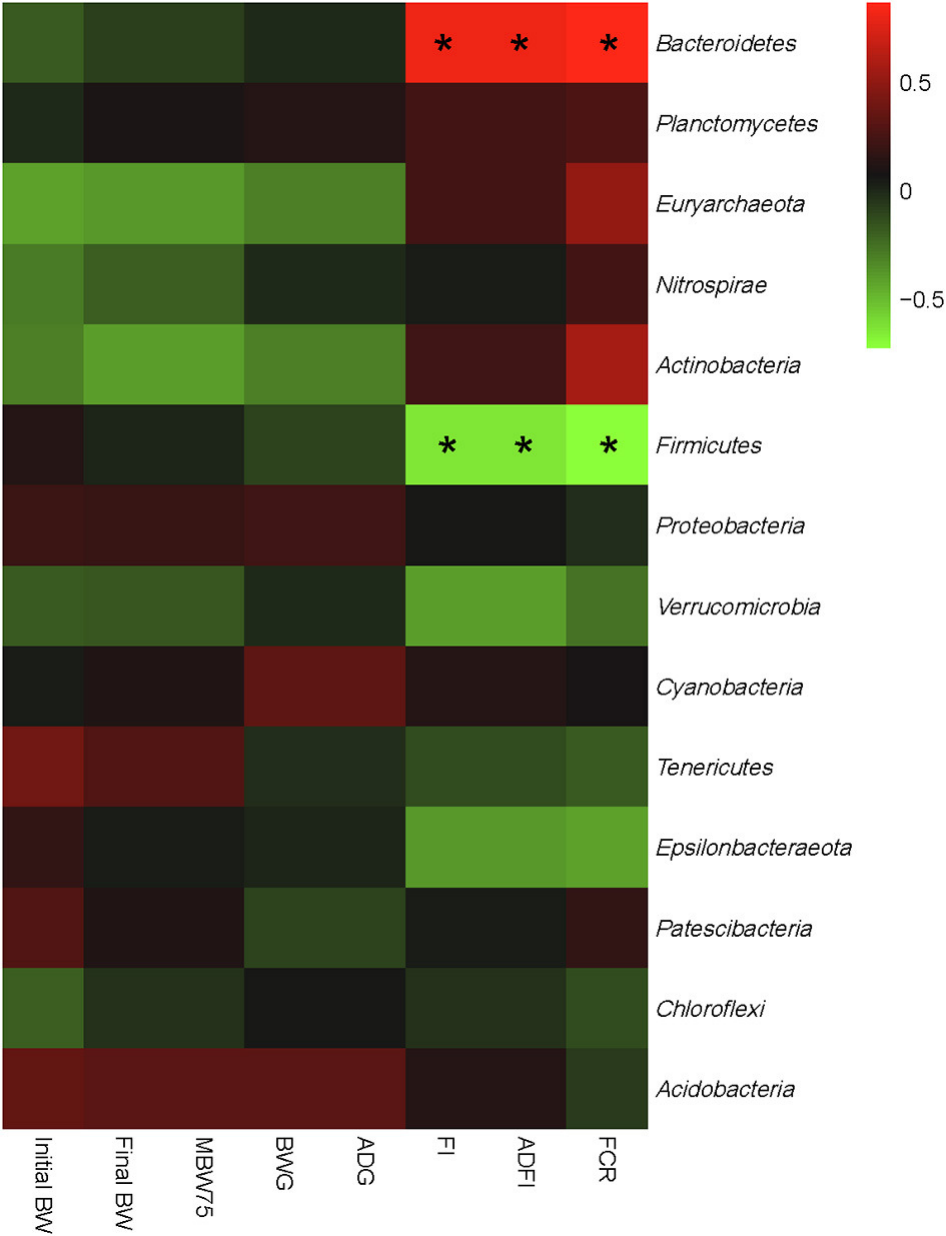
Correlation analysis of relative abundances of cecal bacteria and growth performance of both low- and high-residue feed intake ducks at the phylum level. The red color represents a positive correlation, while the blue color represents a negative correlation. “*” denotes a significant correlation (|r| > 0.55, *P* < 0.05). Initial BW, body weight on day 22; final BW, body weight on day 56; MBW^0.75^, metabolic body weight on day 56; ADG, average daily gain; ADFI, average daily feed intake; FCR, feed conversion ratio; RFI, residual feed intake.

An interactive analysis of gut bacteria and growth performance was conducted, and the results are shown in Figure 4. Integrally, all bacterial phyla could be separated into two large groups: One group mainly consisted of *Bacteroidetes, Planctomycetes, Euryarchaeota, Nitrospirae*, and *Actinobacteria*, which showed a positive correlation with FI, ADFI, and FCR and a negative correlation with BWG, ADG, and MBW^75^. The other group mainly consisted of *Firmicutes, Proteobacteria, Verrucomicrobia, Tenericutes, Epsilonbacteraeota*, and *Acidobacteria*, which showed a completely converse correlation with the growth performance compared with the former group. In particular, *Bacteroidetes* showed a significant positive correlation with FI, ADFI, and FCR (*P* < 0.05), while *Firmicutes* showed a significant negative correlation with the aforementioned growth performance (*P* < 0.05). No other significant correlations were found between the gut microbiota and phenotypic parameters (*P* > 0.05).

### Correlations between the gut microbiota and RFI

**Figure 5:**
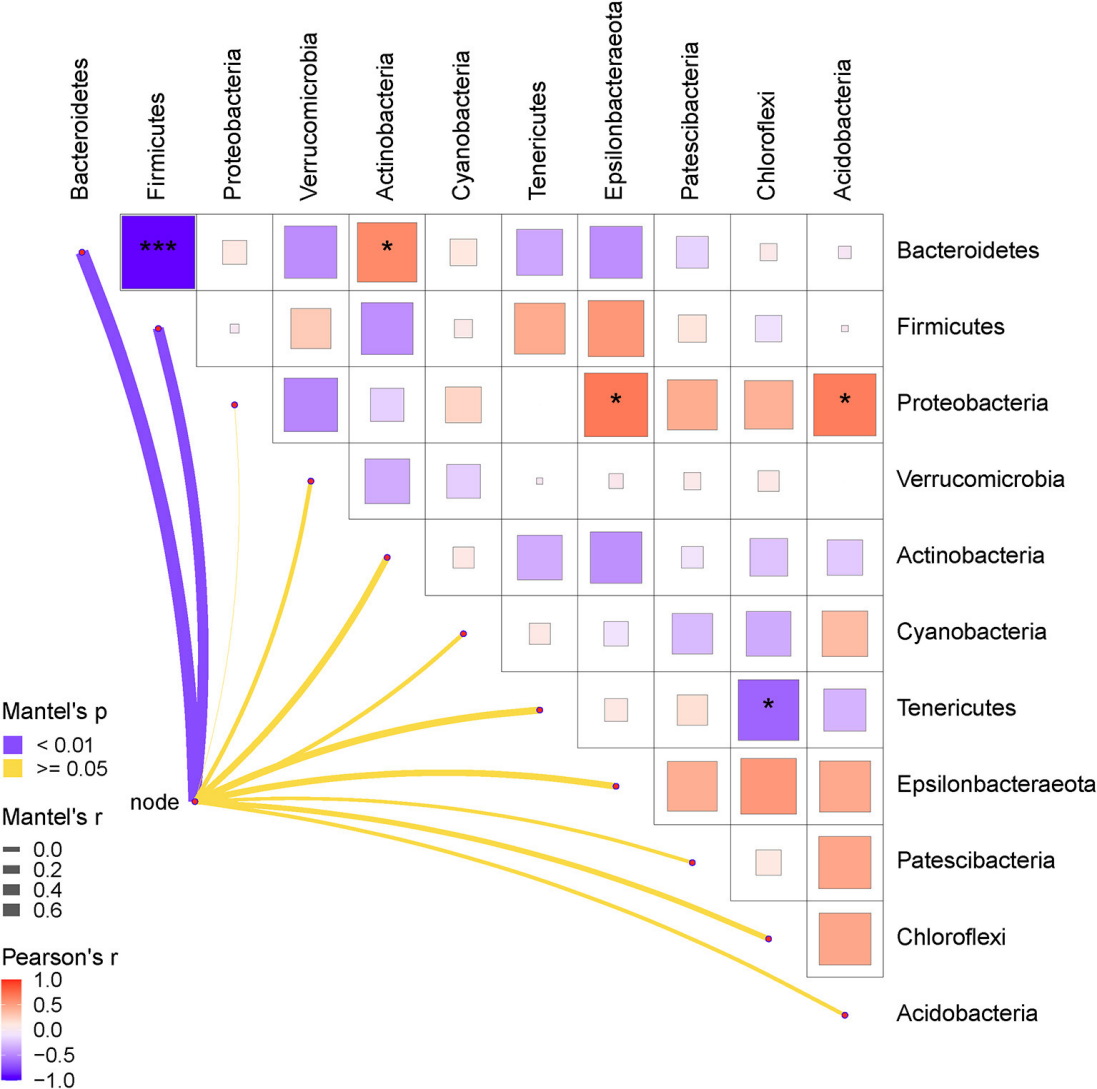
Pearson correlation analysis of the RFI and the relative abundances of cecal bacteria (phyla level). The red color represents a positive correlation, while the blue color represents a negative correlation. The ^*^ symbol denotes a significant correlation (0.35<|r| < 0.55, *P* < 0.05). The ^**^ symbol denotes (0.55<|r|<0.75, *P* < 0.01). The ^***^ symbol denotes (|r| > 0.75, *P* < 0.001).

**Figure 6:**
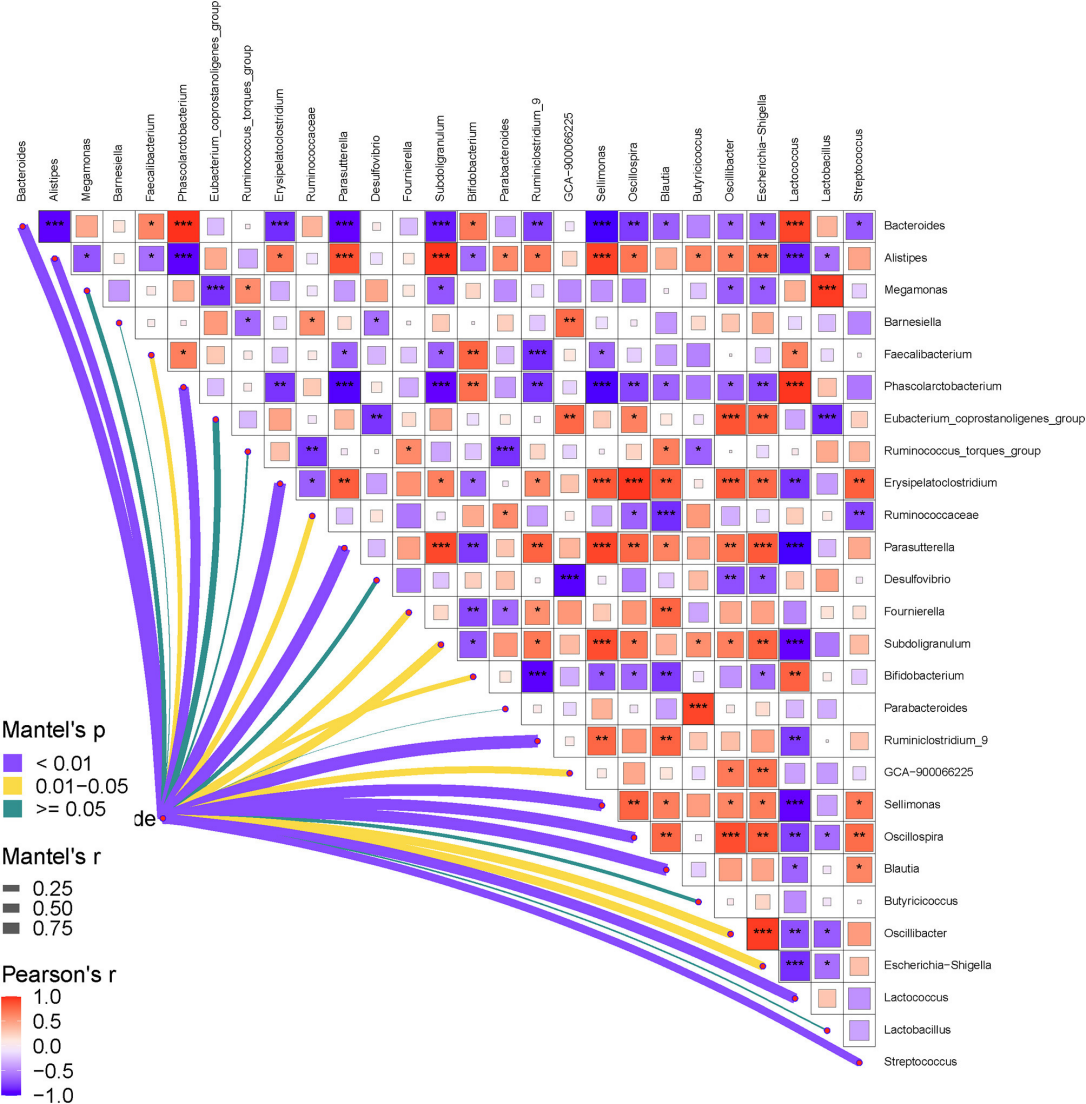
Pearson correlation heatmap analysis of the RFI and the relative abundances of cecal bacteria (genera level). The red color represents a positive correlation, while the blue color represents a negative correlation. The ^*^ symbol denotes a significant correlation (0.35<|r| < 0.55, *P* < 0.05). The ^**^ symbol denotes (0.55<|r|<0.75, *P* < 0.01). The ^***^ symbol denotes (|r| > 0.75, *P* < 0.001).

Finally, the relationships between the gut bacteria and RFI, and the interactive effects among the bacterial communities at both phylum and genus levels were determined. The results are shown in Figures 5, 6. As shown in Figure 5, the RFI showed a significant correlation with *Bacteroidetes* and *Firmicutes* (*P* < 0.05). No other significant correlations were observed between the bacterial phyla and RFI (*P* > 0.05). Specifically, *Bacteroidetes* negatively correlated with *Firmicutes* and synergistically worked with *Actinobacteria* in regulating the RFI. In addition, *Proteobacteria* showed significantly positive correlations with *Epsilonbacteraeota* and *Acidobacteria* (*P* < 0.05), while *Tenericutes* antagonistically worked with *Chloroflexi* in regulating the RFI (*P* < 0.05). No other significant correlations were observed among the bacterial phyla focused on RFI regulation (*P* > 0.05). Further analysis of the bacterial genera and RFI was performed, and the results are shown in Figure 6. Integrally, the RFI was significantly correlated with *Bacteroides, Alistipes, Phascolarctobacterium, Erysipelatoclostridium, Parasutterella, Fournierella, Subdoligranulum, Bifidobacterium, Ruminiclostridium_9, Sellimonas, Oscillospira, Blautia, Oscillibacter, Escherichia-Shigella, Lactococcus*, and *Streptococcus* (*P* < 0.05). Specifically, the most abundant genus *Bacteroides* showed a significant negative correlation with *Alistipes, Erysipelatoclostridium, Parasutterella, Subdoligranulum, Ruminiclostridium_9, Sellimonas, Oscillospira, Blautia, Oscillibacter, Escherichia-Shigella*, and *Streptococcus* (*P* < 0.05), while it showed a significantly positive correlation with *Phascolarctobacterium, Bifidobacterium*, and *Lactococcus* (*P* < 0.05). Moreover, *Faecalibacterium* synergistically regulated the RFI with *Bifidobacterium* and *Lactococcus* and antagonistically worked with *Parasutterella, Subdoligranulum, Ruminiclostridium_9*, and *Sellimonas* (*P* < 0.05). *Escherichia-Shigella* mainly showed a negative correlation with the RFI and a negative correlation with *Bacteroides, Megamonas, Phascolarctobacterium, Desulfovibrio*, and *Bifidobacterium* (*P* < 0.05). Probiotics such as *Bifidobacterium* and *Lactococcus* showed a synergistic correlation with *Bacteroides, Faecalibacterium*, and *Phascolarctobacterium* (*P* < 0.05), while they antagonistically worked with *Alistipes, Erysipelatoclostridium, Parasutterella, Fournierella*, and *Subdoligranulum* (*P* < 0.05).

## Discussion

The selection of low-RFI animals effectively reduces feed consumption and increases the income generated from chicken, steer, and swine production. Similarly, our study evaluated the relationship between the FE and the RFI index, uncovered a 60-g reduction in the ADFI of ducks of similar weights in both LR and HR groups, which further indicated a significant reduction of feed costs and an increase of productive profit for industrial duck producers. This result validated the rationality of our selection of LR and HR ducks.

### Correlations between the gut microbiota and nutrient digestibility

Nutrient digestibility is significantly modulated by both the gastrointestinal microbiota communities and the interactive effects between bacteria and the host intestinal epithelial proteins (Rowland et al., 2018; Zhao et al., 2019). In general, more than 90% of the gut microbial profiles belong to *Firmicutes* and *Bacteroidetes* (Tremaroli and Backhed, 2012), which was also found in the present study. The ratio of *Firmicutes* to *Bacteroidetes* is positively correlated with energy metabolism, which indicates that a higher *Firmicutes* abundance boosts starch digestibility, and thus, more energy is provided (Tremaroli and Backhed, 2012). In this study, *Firmicutes* significantly increased in LR ducks compared with HR ducks, and the proliferated *Firmicutes* may help to promote the degradation of feed concentrates and to improve the FE. Furthermore, increased amounts of probiotics may also help to maintain intestinal homeostasis and thereafter promote nutrient utilization. Specifically, *Bifidobacterium*, which plays an important role in maintaining intestinal homeostasis (Luo et al., 2018; Engevik et al., 2019), was significantly proliferated in LR ducks. This alteration helped to re-establish the gut microbial communities and modulated the interactions among bacterial communities, which further improved the nutritional digestibility and FE. Moreover, the increase in *Bifidobacterium* closely correlated with adiposity and microbe-derived inflammatory molecule reduction (Ramirez-Farias et al., 2009). Such an increase after the increment of *Bifidobacterium* protected the intestinal structure, enhanced intestinal barrier functions, and further promoted the nutrient intestinal absorptivity.

### Relationship between the gut microbiota and RFI

In broad terms, there are likely to be at least four major processes that impacted FE, namely, FI, feed digestion, anabolism, and catabolism (Bezerra et al., 2013; Soleimani and Gilbert, 2020; Martin et al., 2021). Previous studies highlighted that animals with a lower RFI generally showed enhanced bacterial degradability, higher carbohydrate metabolism, and activated enzymatic catalytic capacity (Liu et al., 2021, 2022). In the present study, the RFI was significantly correlated with *Bacteroides, Bifidobacterium, Ruminiclostridium_9, Sellimonas, Escherichia-Shigella, Lactococcus*, and *Streptococcus*, which actively participated in physiological carbohydrate metabolism and may have provided further energy for body growth. Furthermore, genes that participated in the volatile fatty acid absorption process, such as *SLC16A3, SLC26A3*, and *HIF1A*, and those that clustered into ketogenesis including *LDHA, LDHB*, and *FFAR2* were proved to be highly positively correlated with a lower RFI (Salleh et al., 2017). These findings indicate that bacteria that participated in volatile fatty acid and ketosis metabolism processes may interact with the host genes, which ultimately proves the close correlation between the gut microbiota and RFI.

### The underlying regulatory pattern of gut microbiota on the RFI

Nowadays, research focusing on the functions of gut microbiota is growing vigorously. The regulatory effects of gut microbiota on enteritis, cardiovascular disease, fecal microbiota transplants, and the subsequent interactions with host genes are well-studied. However, it is a challenge to discern general principles explaining the diversity and dynamics of complex multi-species communities, since detailed biological parameters are not typically available in large ecological networks (Krumbeck et al., 2021). As the body is a complicated system, the regulatory mechanism of the gut microbiota on the RFI also presents a systematic coexistence and a tunable interactive effect, as shown in Figures 3, 4. Bacterial communities can synergistically or antagonistically interact with one another and can ultimately determine nutrient digestibility and absorption. Traditionally, bacteria with similar degradative orientations synergistically interact with one another to further promote nutrient digestion, for example, *Bifidobacterium* and *Lactococcus* in carbohydrate metabolism (Fernández et al., 2020). Besides interactiona within bacterial communities, the morphogenesis effects of gut microbiota on body intestinal epithelial development through secondary metabolites such as butyrate also work to regulate the FE (He et al., 2021). To date, host and bacterial interactions are a great causal factor that can shape the gastrointestinal tract, impact growth rates, and carry capacity by providing more energy and scavenging inflammatory molecules (Jackrel et al., 2020; Houwenhuyse et al., 2021). Gut microbiota can regulate intestinal development by acquiring indispensable materials from degrading carbohydrates and proteins. Nutrient absorption occurs in the gastrointestinal tract and is regulated by high levels of acids, abundant digestive enzymes, and antimicrobials (O'Callaghan and Corr, 2019). The epithelium also contains plentiful functional proteins, which conduct substance interchange or achieve information exchange with other target metabolites. Intriguingly, microbial communities that positively correlate with the RFI are mainly clustered into carbohydrate-degraded organisms; therefore, more effective energy utilization is provided for body growth.

In summary, the RFI was significantly correlated with gut microbial communities and was regulated by the interactive effects between the gut microbiota and crosstalk with the host genes. These microbiota communities related to carbohydrate metabolism have positive regulatory effects on the low-RFI ducks. In future, we aim to improve the relative abundance and utilization of these communities to promote the FE of small-sized meat ducks. The present study provides valuable insights into the dynamics of gut microbiota underlying the variations in the FE of small-sized meat ducks.

## Data availability statement

The datasets presented in this study can be found in online repositories. The names of the repository/repositories and accession number(s) can be found in the article/Supplementary material.

## Ethics statement

The animal study was reviewed and approved by the Animal Care and Use Committee of Yangzhou University (No. SYDW-2019015).

## Author contributions

GCha, FX, and HB conceived and designed the experiments. LS, QG, YJ, XL, DG, and CW performed the experiments and participated in the data collection. HB and FX analyzed the 16S rRNA data and wrote the manuscript. HB, FX, YB, ZW, GChe, and GCha revised the manuscript. All authors contributed to the article and approved the submitted version.

## Supplementary material

Supplementary Table 1 Taxonomy results of the gut microbiome of each sample.
